# Field Deployment Strategies for UAV-Based Chemical Sensors: Systematic Evaluation of Direct Drop, Tethered Hovering, and Low-Altitude Placement in Hazardous Chemical Scenarios

**DOI:** 10.3390/s26216985

**Published:** 2026-11-05

**Authors:** Wen Yang, Yelin Qi, Hao Wen, Yutong Pan, Ting Liang

**Affiliations:** Institute of NBC Defence PLA ARMY, Beijing 102205, China

**Keywords:** unmanned chemical sensor, downwash flow mitigation, deployment strategy, field validation, chemical threat scenario, sensor survival rate, unmanned ground vehicle

## Abstract

Unmanned aerial vehicles (UAVs) and unmanned ground vehicles (UGVs) equipped with chemical sensors hold great promise for hazardous incident reconnaissance, yet practical deployment is hindered by sensor survival upon impact, rotor downwash interference, and terrain-induced trade-offs between accuracy and efficiency. In this study, we focus on UAV-based deployment but also consider UGVs as complementary platforms for ground-level placement and long-term monitoring. To address these challenges for typical operational scenarios, we first implemented two customized engineering improvements: a carbon-fiber anti-downwash plate and a reinforced tether–release mechanism. Using a custom hexacopter with a 1 kg sensor prototype, we then systematically evaluated three deployment strategies. Direct free-fall from 5 m resulted in 0% survival due to housing fracture and circuit damage. The anti-downwash plate reduced local airflow by 70%, establishing a 6 m safe tether length. Tethered hovering achieved 100% survival, ~3 min/point mission time, and strong terrain adaptability, albeit with moderate dispersion. Low-altitude gentle placement delivered the highest accuracy and 100% survival but required 8 min/point and flat terrain. Based on these experimental results, we derive a mission-adaptive decision matrix for four representative chemical scenarios, recommending tethered hovering as the primary strategy for complex terrains, low-altitude placement for fixed long-term monitoring on flat surfaces, and direct drop only if cushioning is added and height is reduced to ≤3 m. We further discuss how the decision matrix can be extended to multi-sensor UAVs, UGVs, and UAV–UGV collaborative systems. Our findings offer a practical, evidence-based operational framework tailored to unmanned chemical reconnaissance.

## 1. Introduction

The rapid detection and mapping of airborne chemical hazards—arising from industrial accidents, terrorist attacks, leakage of hazardous chemicals, or hazardous material transport incidents—are critical for emergency response, civilian protection, and defense operations. Traditional ground-based chemical detection is often slow, exposes personnel to danger, and provides limited spatial coverage. Unmanned aerial vehicles (UAVs) and unmanned ground vehicles (UGVs) equipped with miniaturised chemical sensors have emerged as a transformative solution, offering advantages in safety, mobility, and wide-area rapid reconnaissance [1].

Over the past decade, significant progress has been made in sensor miniaturisation, with various platforms demonstrating the ability to detect volatile organic compounds (VOCs), ammonia, hydrogen sulphide, and toxic industrial chemical simulants [2,3,4,5,6,7,8]. UAVs have been widely used for aerial mapping of chemical plumes, while UGVs offer complementary capabilities for ground-level sampling, confined-space access, and long-duration monitoring. However, translating laboratory-ready sensors into operational field deployment remains fraught with practical challenges that are often overlooked in sensor-centric research [9,10]. Three critical barriers stand out.

First, sensor survival upon ground impact has rarely been quantified. Many unmanned deployment concepts rely on releasing sensors to the ground—either for long-term monitoring or because the UAV cannot land. A typical 1 kg sensor dropped from a height of 5 m acquires a kinetic energy of approximately 49 J. The ABS enclosure used in this study had a wall thickness of 2.5 mm and dimensions of approximately 120 mm × 80 mm × 60 mm. The peak impact stress on the thin ABS walls exceeds the material’s notched impact strength (typically 200–400 J/m) and tensile strength (≈40–50 MPa), leading to brittle fracture. Without adequate cushioning, internal components are prone to fracture or detachment. Existing studies on airdrop packaging focus on heavy military equipment or use bulky foam enclosures that are incompatible with the weight and size constraints of UAV payloads. A systematic assessment of failure modes and mitigation strategies is urgently needed.

Second, rotor downwash interference can severely distort local gas concentrations. The high-velocity turbulent airflow generated by UAV propeller downwash has been shown to dilute or perturb gas samples, leading to artificially low or fluctuating readings [11,12]. Common mitigation strategies include mounting the sensor on a long pole extending laterally from the UAV or using a tether to lower the sensor away from the rotors [13]. However, long poles increase inertia and reduce flight stability, while tethers are susceptible to pendulum effects and entanglement. A lightweight, passive downwash mitigation device that can be easily integrated into existing UAVs and UGVs is desirable but has not been systematically characterised.

Third, trade-offs between deployment accuracy, speed, and terrain adaptability are not well understood. Different mission contexts demand different priorities. For example, pinpoint placement of a sensor into a manhole or a specific leak location requires centimetre-level accuracy, which may come at the cost of mission time (slow descent). Conversely, for wide-area search missions, rapid deployment (e.g., free drop) is prioritised, but accuracy and sensor survival may be compromised. Furthermore, real-world terrains are rarely flat: industrial accident sites are strewn with rubble, urban environments contain curbs and vegetation, and transport accidents occur on slopes and ditches. UGVs can operate on some flat or semi-flat surfaces but face challenges on steep slopes, rubble, and confined spaces. The compatibility of each deployment strategy with such complex terrains has not been quantitatively compared [14,15,16].

A head-to-head, quantitative field evaluation of the three fundamental deployment strategies—direct free-fall drop, tethered hovering with downwash mitigation, and low-altitude gentle placement—using the same UAV platform and sensor prototype under controlled but realistic conditions is therefore required [17,18]. Furthermore, translating such experimental findings into actionable operational guidelines for hazardous chemical scenarios represents a critical step toward practical deployment [18,19]. In this work, we deliberately use a single-sensor, single-platform configuration as a controlled baseline to isolate the effect of the deployment method. We also discuss how the framework can be extended to multi-sensor UAVs and UAV–UGV collaborative systems [19,20].

In this work, we aim to fill this gap by: (i) developing an EHM-1500 hexacopter platform integrated with a winch-based tether system and a carbon-fibre anti-downwash plate; (ii) conducting systematic field experiments on concrete and grass surfaces to measure sensor survival rate, deployment accuracy, downwash resistance, and mission time for each strategy; (iii) using computational fluid dynamics (CFD) simulations to map downwash velocity fields and guide the design of the anti-downwash plate; (iv) analysing damage modes after free fall and proposing engineering improvements; (v) comparing the three strategies based on multiple dimensions (survival, accuracy, downwash resistance, mission time, terrain adaptability, reusability); and (vi) mapping the results onto four typical hazardous chemical scenarios to produce a mission-adaptive decision matrix. We also outline how the decision matrix can be extended to UGVs and multi-sensor unmanned systems. Our findings provide an evidence-based operational framework for unmanned chemical reconnaissance and highlight design guidelines for next-generation sensor packaging and unmanned platform integration.

## 2. Materials and Methods

### 2.1. UAV Platform and Sensor Prototype

The UAV platform was an EHM-1500 hexacopter (Electric Eagle Technology Co., Ltd., Wuhan, China) (as shown in Figure S1) with the following specifications: maximum payload of 8 kg, endurance of 40 min, RTK-GPS horizontal accuracy of ±0.1 m, maximum wind resistance of up to Beaufort scale 6, IP54 rating, and communication range of 20 km. The platform’s capability profile (normalised radar chart, as shown in Figure 1) met or exceeded all military-relevant thresholds defined in Chapter 2 of our companion study.

The chemical sensor was a custom-assembled prototype weighing 1 kg, enclosed in an ABS plastic housing. It was not a commercial off-the-shelf product; rather, it was developed by the authors to represent a realistic payload for unmanned chemical reconnaissance. It contained a gas detection module (electrochemical ammonia sensor), a rechargeable battery, a power switch, and a 4G wireless data transmission unit. The power switch was manually operated before each test; it was not controlled remotely. The 4G unit was used only for real-time data downlink. Prior to each test, sensors were charged and verified for normal data transmission. The same sensor type was used in all deployment tests (DD, TH, LP) to ensure consistency.

### 2.2. Field Test Site and Meteorological Conditions

All field experiments were conducted at an open-air test facility in Wuhan, China. The test area included two distinct surface types: (i) a concrete hardstand and (ii) a natural grassland. The wind speed ranged from 1.5 to 3.5 m/s, the air temperature ranged from 15 to 20 °C, and the relative humidity ranged from 45% to 55%; there was no precipitation. These conditions are typical for spring/autumn in central China and are within the safe operating envelope of the UAV. A handheld weather station was used to record conditions every 30 min.

### 2.3. Deployment Strategies: Procedures

Three deployment strategies were defined and systematically tested. Each strategy was designed to address a distinct operational need: rapid wide-area coverage (direct drop), interference-free vertical profiling (tethered hovering), or high-precision fixed-point placement (gentle placement). The procedures are detailed below.

A. Direct free-fall drop (DD)

This strategy emulates the simplest and fastest deployment mode, intended for scenarios where sensor reusability is not required and terrain conditions are permissive. The UAV ascended to a predetermined hover height above the target point, with the carrier plate heights set at 3 m, 5 m, 8 m, and 10 m to evaluate the influence of drop height on impact survival and landing accuracy. Upon stabilisation, a 4G remote command triggered the release mechanism, allowing the sensor to fall freely to the ground without any cushioning. Each height-surface combination (concrete and grass) was repeated 3–5 times, with five drops conducted at the baseline height (two on concrete, three on grass) and additional repetitions for the other heights.

B. Tethered hovering (TH)

This strategy was developed to eliminate rotor downwash interference while enabling vertical profiling of gas concentrations at the breathing zone (≈1.5 m above ground). The UAV first climbed to a safe hover height of ≥6 m, a threshold determined by prior downwash characterisation. A winch system then lowered the sensor at a constant speed of 0.5 m/s to the desired sampling height. The sensor was kept at that altitude for 10–30 s to obtain stable readings, after which it was winched back. The carbon-fibre anti-downwash plate was permanently installed in this mode to deflect the rotor-induced airflow laterally, creating a low-velocity quiet zone around the sensing element.

To quantify the influence of tether length on landing accuracy (for potential ground-contact operations), a separate series of tests was performed under the same low-wind conditions (1–2 m/s). The sensor was lowered until it gently touched the grass surface (soft contact), then released. Three tether lengths—5 m, 8 m, and 14 m—were evaluated, with five repetitions per length.

C. Low-altitude gentle placement (LP)

This strategy aimed to achieve the highest possible placement accuracy while preserving sensor integrity, albeit at the cost of longer mission time and strict terrain requirements. The UAV descended vertically from a safe height at a speed ≤0.5 m/s. Descent continued until ground contact was reliably detected by the UAV’s downward-facing sonar altimeter and visually confirmed via a 4G camera. Only then was the release command sent, and the UAV immediately climbed back to its original altitude. This procedure mimics “planting” the sensor on the ground without any free-fall impact. All LP tests were conducted on flat, obstacle-free surfaces to avoid tilting or damage. Mission time was recorded from takeoff to the moment the UAV returned to its initial safe altitude after sensor release.

For all three strategies, the same sensor payload and UAV platform were used. Each test was repeated a minimum of three times unless otherwise noted, and the order of tests was randomised to minimise systematic errors.

### 2.4. Evaluation Metrics

Sensor survival rate was defined as the percentage of sensors that remained structurally intact and able to transmit 4G data post-impact. Damage was classified into four categories for failure mode analysis: shell fracture, power switch detachment, circuit board fracture, and battery dislodgement.

Deployment accuracy was quantified as the dispersion diameter. Landing coordinates were measured using a high-precision GPS.

Downwash resistance was evaluated by comparing sensor readings near a controlled ammonia evaporation source. The sensor’s baseline reading was 360 ± 5 ppm. The UAV then hovered at altitudes of 4 m, 5 m, 6 m, and so on, with and without the anti-downwash plate, while the sensor was positioned at a fixed height of 1.5 m above the source. The maximum relative deviation from baseline was recorded [21].

Mission time was measured using a stopwatch from takeoff to the moment the first stable sensor data were received at the ground control station after deployment, and the UAV had returned to a safe altitude. For DD, this included only the time to reach hover height, release, and climb away; for TH, it included winch lowering, dwell, and winch retrieval; for LP, it included the slow descent and climb.

### 2.5. Computational Fluid Dynamics (CFD) Simulation

To understand the spatial distribution of downwash velocity, we performed 3D steady-state Reynolds-averaged Navier–Stokes (RANS) simulations using ANSYS Fluent 2024. The UAV geometry was placed in a computational domain of 10 m × 10 m × 10 m. Rotor speed was set to 2200 RPM, corresponding to a thrust of 2.5 kg per rotor. The downwash velocity magnitude was sampled on vertical planes passing through the rotor axis. The mesh had approximately 4 million tetrahedral cells with inflation layers near the ground. Turbulence was modelled using the realizable k-ε model with enhanced wall treatment. Boundary conditions: ground as no-slip wall, sides and top as pressure outlets [22,23]. The CFD simulations were used because downwash flow is difficult to measure experimentally in all spatial locations; CFD provides a cost-effective way to map velocity fields and optimize the anti-downwash plate design.

### 2.6. Data Analysis

All statistical analyses were performed using Python (3.11.5) with libraries SciPy and statsmodels. For accuracy comparison across different tether lengths, one-way ANOVA was used after checking normality and homogeneity of variances. For downwash tests, paired *t*-tests were applied to compare readings with and without the anti-downwash plate at each altitude. Significance was set at α = 0.05.

## 3. Results

### 3.1. Direct Free-Fall Drop

#### 3.1.1. Sensor Survival and Failure Modes

For the direct free-fall drop strategy, sensor survival upon ground impact is the most critical performance metric, as a damaged sensor cannot provide reliable data and may be unrecoverable [24]. To assess survival under different landing surfaces, sensors were dropped from a hover height of 5 m onto concrete and grass. When the sensor was released from a hover height of 5 m, a 0% survival rate was observed (n = 5). On concrete surfaces (n = 2), both sensors sustained catastrophic damage, including complete shell rupture, power switch detachment, circuit board fracture, and solder joint failure (as shown in Figure 2). On grass (n = 3), two sensors were severely damaged, and one exhibited minor cracks but required reassembly before it could function; therefore, it was also classified as severe damage. The quantitative distribution of damage severity is shown in Figure 3: concrete resulted in 100% severe damage, whereas grass yielded 100% severe damage after reclassification.

Failure mode analysis across the five sensors (as shown in Figure 4) revealed that shell fracture and power switch detachment each accounted for 29% of the total 17 failure incidences, followed by circuit board cracking (24%) and battery dislodgement (18%). The impact kinetic energy was calculated as 49. The ABS enclosure had a wall thickness of 2.5 mm. The peak impact stress on the thin walls exceeded the material’s notched impact strength (typically 200–400 J/m) and tensile strength (≈40–50 MPa), explaining the observed structural failures. A finite element analysis (FEA) simulation showed that the maximum von Mises stress at the shell corners reached ~85 MPa at a 5 m drop, well above the ABS yield strength.

#### 3.1.2. Effect of Drop Height on Landing Accuracy

Beyond sensor survival, the precision of landing is another key concern for direct drop, as a large dispersion may miss the intended target. To quantify how drop height influences landing accuracy, additional tests were conducted at heights of 3 m, 8 m, and 10 m, in addition to the baseline 5 m. Deployment accuracy, quantified as the dispersion diameter, increased with drop height for both surface types (as shown in Figure 5). On concrete, the dispersion rose from 0.3 m at 3 m height to 1.2 m at 10 m (slope = 0.13 m/m). On grass, the increase was steeper (from 0.5 m to 1.9 m; slope = 0.18 m/m), attributable to terrain-induced bouncing and rolling. These results indicate that direct free-fall drop is not feasible for operational use without additional impact-absorbing measures.

### 3.2. Tethered Hovering with Downwash Mitigation

#### 3.2.1. Anti-Downwash Modification: Design and Installation

The downwash flow generated by the UAV’s rotors is a critical source of interference for airborne gas sensors [25]. During hover, the high-speed rotation of the propellers creates a strong turbulent airflow directly beneath the airframe. If the sensor is exposed to this flow, the local gas concentration is rapidly diluted and the measurement becomes severely distorted. Computational fluid dynamics (CFD) simulations (as shown in Figure 6) showed that, under full-load conditions, the airflow velocity at the sensor position could reach 13 m/s.

To mitigate this effect, a carbon-fibre anti-downwash plate (as shown in Figure 7) was developed. Its working principle is to place a physical barrier above the sensor that deflects the downwash laterally, creating a low-velocity stable zone around the sensing element.

The plate was fabricated from T700-grade carbon-fibre pre-preg using a quasi-isotropic layup, compression-moulded to a thickness of 1.5 mm. Its circular shape, with a diameter of 380 mm (approximately 1.18 times the rotor disk diameter of 320 mm), ensures consistent flow redirection from any azimuth and avoids stress concentration points. The plate has a mass of approximately 50 g and a 20-mm-diameter hole at its centre for the tether to pass through (as shown in Figure S2). It was connected to the lower carrier plate by four stainless-steel suspension rods (3 mm diameter, 150 mm length, threaded at both ends). The vertical distance between the anti-downwash plate and the sensor was set to 120 mm, which was optimised by CFD simulations to allow sufficient space for flow expansion and deceleration while minimising pendulum-induced lateral displacement.

A custom-designed winch deployment system was attached to the UAV’s underside. It consisted of the anti-downwash plate, an electric winch with a maximum tether length of 20 m, and a 4G-controlled release mechanism. The winch allowed controlled lowering and retrieval of the sensor at speeds of 0.2–1.0 m/s.

#### 3.2.2. Validation of Downwash Suppression

The effectiveness of the anti-downwash plate was evaluated through both computational fluid dynamics (CFD) simulations and field experiments using an ammonia release source. CFD simulations showed that without the plate, the downwash velocity at the sensor location reached up to 13 m/s under full-load hover conditions. With the plate installed at an optimal spacing of 120 mm, the simulated airflow velocity in the sensor region was reduced to below 4 m/s, representing a reduction of approximately 70%.

Experimental validation was performed using a controlled ammonia evaporation source (10% aqueous ammonia, 100 mL in a 20 cm diameter dish). The reading was obtained with the same custom-assembled chemical sensor prototype used in all deployment tests. The gas detection module is an electrochemical ammonia sensor. The ground-level baseline reading was 360 ± 5 ppm (mean ± SD, n = 10). As shown in Figure 8, without the plate, the sensor reading remained near baseline from ground level up to 4 m hover height. At 5 m, however, the reading spiked sharply to 523 ppm—a 45% relative increase—before returning to baseline at 6 m and above. This anomalous spike is attributed to the high-velocity downwash entraining ammonia vapour from the source and accelerating it towards the sensor, which would cause false positives in real threat scenarios.

With the anti-downwash plate installed, the sensor was lowered via tether from a 6 m hover to 1.5 m above ground. Throughout the descent, readings remained stable between 358 and 365 ppm, with no spikes or oscillations. The plate effectively redirected the downwash flow laterally, creating a low-velocity “quiet zone” underneath. These experimental results, consistent with the CFD predictions, confirm that the plate completely eliminates downwash-induced measurement distortion. Based on these findings, a minimum safe tether length of 6 m was established for all subsequent tethered hovering operations.

#### 3.2.3. Effect of Tether Length on Landing Accuracy

For operations where the tethered sensor may need to be placed on the ground (e.g., for long-term monitoring), the effect of tether length on landing accuracy must be understood. Under low-wind conditions (1–2 m/s), tether length had no statistically significant effect on landing dispersion (one-way ANOVA, *p* = 0.38). Figure 9 presents the landing points in polar coordinates for tether lengths of (a) 5 m, (b) 8 m, and (c) 14 m. The dispersion is centered around the target point, with no systematic drift observed. The overlapping distributions indicate that tether length does not significantly affect landing accuracy under low-wind conditions. The corresponding box-and-whisker plot (Figure 10) showed mean dispersion diameters of 1.8 m, 1.9 m, and 1.6 m, respectively, with overlapping interquartile ranges. The dominant factor was the UAV’s RTK-GPS hover accuracy (±0.1 m), while pendulum effects were negligible at these wind speeds.

### 3.3. Low-Altitude Gentle Placement

#### 3.3.1. Sensor Survival and Deployment Accuracy

The low-altitude gentle placement strategy prioritises sensor integrity and precise positioning over speed. Therefore, survival rate and landing accuracy were the primary metrics evaluated. In this method, the UAV descended vertically at ≤0.5 m/s until ground contact was detected, and then released the sensor. Across six trials (three on concrete, three on grass), all sensors survived (100% survival rate), with no structural damage or data transmission failure. The landing dispersion diameter was ≤0.2 m, the highest accuracy among the three strategies. This sub-decimetre precision makes low-altitude placement ideal for tasks requiring exact sensor positioning, such as placement inside a manhole or onto a specific valve. UGVs can also perform this task on flat surfaces, providing an alternative when UAV operation is restricted.

#### 3.3.2. Mission Time and Terrain Sensitivity

Although the gentle placement method offers perfect survival and high accuracy, its operational efficiency and terrain adaptability may limit its use. To quantify these limitations, mission time and terrain sensitivity were examined. The mean mission time was 8.0 ± 0.5 min per point, substantially longer than direct drop (2.0 ± 0.2 min) and tethered hovering (3.0 ± 0.3 min) (Figure 11). The slow descent and ascent accounted for over 5 min of this time. Moreover, the method proved highly sensitive to terrain flatness. On slightly uneven surfaces (e.g., grass with small rocks or cracked asphalt), the sensor often tilted or failed to stand upright, compromising measurement reliability. On surfaces with hidden obstacles, minor shell damage occurred in preliminary tests.

### 3.4. Overall Comparison of the Three Deployment Strategies

To enable a holistic assessment, the three deployment strategies were compared across six quantitative metrics: sensor survival rate, deployment accuracy (inverse of dispersion diameter), downwash resistance (reading stability), mission time (inverse of minutes per point), terrain adaptability, and reusability. All metrics were normalised to a 0–1 scale, where 1 represents the best performance among the three strategies.

A parallel coordinates plot (Figure 12) and a Nightingale rose diagram (Figure 13) visualise the multi-dimensional trade-offs. Tethered hovering achieved the highest composite scores: survival = 1.0, downwash resistance = 0.9, terrain adaptability = 0.9, and reusability = 0.9, with a moderate mission time score (0.6) and accuracy score (0.15). Low-altitude placement excelled in survival (1.0) and accuracy (0.9) but scored poorly in mission time (0.2) and terrain adaptability (0.3). Direct drop performed worst overall: survival = 0.0, downwash resistance = 0.5, reusability = 0.2, and terrain adaptability = 0.4.

Based on these comparisons, a mission-adaptive decision matrix was constructed (Figure 14). For four typical chemical threat scenarios (industrial accident, terrorist attack, warfare agent leakage, and transport accident), tethered hovering was found to be the most versatile and reliable strategy, being recommended (Y) for all scenarios where terrain is uneven or rapid sensor reusability is required. Low-altitude placement is optional (O) only for fixed-point long-term monitoring on flat surfaces. Direct drop is not recommended (N) for any scenario unless future lightweight cushioning is developed and validated. The matrix can be extended to include UGVs for flat-surface placement and multi-sensor UAVs for wide-area coverage.

## 4. Discussion

### 4.1. Sensor Impact Resistance and the Infeasibility of Direct Drop

The 0% survival rate observed for direct free-fall drop from 5 m height is attributed to a fundamental mismatch between the impact kinetic energy (≈49 J) and the limited impact toughness of the ABS housing. The ABS enclosure had a wall thickness of 2.5 mm. The peak impact stress on the thin walls exceeded the material’s notched impact strength (typically 200–400 J/m) and tensile strength (≈40–50 MPa). A finite element analysis (FEA) simulation showed that the maximum von Mises stress at the shell corners reached ~85 MPa at a 5 m drop, well above the ABS yield strength. The dominant failure modes—shell fracture and power switch detachment—indicate that the outer casing and its integrated components represent the weakest structural links.

#### 4.1.1. Advanced Enclosure Design for Improved Survival

To improve the survival probability of the sensor in future direct-drop operations, we propose and evaluate several advanced enclosure designs. Table 1 summarizes the estimated survival probability and mass penalty for each option.

#### 4.1.2. Engineering Improvements for Direct Drop

To enable future use of direct drop in operational settings, three engineering improvements are proposed.

First, lightweight cushioning materials, such as 10 mm expanded polypropylene (EPP) foam, microcellular rubber, or degradable airbags, can be wrapped around the sensor to absorb impact energy through plastic deformation or gas compression. Such buffers have been shown to reduce peak acceleration from >1000 g to <200 g in similar drop tests for portable electronics. The mass penalty of a 10 mm EPP layer is approximately 50 g for a 1 kg sensor, which is acceptable for most UAV payloads. Second, a modular internal suspension system can be implemented, wherein the printed circuit board (PCB) and battery are elastically mounted inside a rigid shell. Under impact, the outer shell absorbs the initial blow and may crack, but the inner floating assembly decelerates more gradually, preserving critical electronic connections. Third, for larger or more expensive sensors, an active airbag system could be integrated: a miniature accelerometer detects free-fall and triggers a gas generator to inflate an airbag milliseconds before ground impact. In addition, a controlled-opening package box could remain closed during free fall and open after landing to expose the sensor and activate it remotely. While technically more complex, this approach has been successfully demonstrated in drone-delivered payloads and could simultaneously solve the survival-versus-weight dilemma.

Until such improvements are validated through further drop testing, direct drop should be used only under very strict constraints: drop height ≤3 m and landing on soft, deformable terrain. In all other cases—especially on hard surfaces or when sensor reusability is required—direct drop is not recommended.

### 4.2. Downwash Mitigation and the Role of Tether Length in Landing Accuracy

The carbon-fibre anti-downwash plate proved highly effective, reducing the local airflow velocity at the sensor position by approximately 70% (from 13 m/s to <4 m/s) and completely eliminating the severe reading spike observed without the plate. This design is not limited to the present sensor-UAV combination; it can serve as a universal accessory for any multi-rotor UAV carrying a gas sensor. The key geometric parameters—plate diameter ≥ 1.2 × rotor disk diameter, vertical gap of 10–20 cm between the rotor plane and the plate, and a sensor located at least 5 cm below the plate—can be adjusted for different airframes. The plate adds negligible weight (<50 g) and does not create appreciable drag during forward flight. It is recommended that such a plate be adopted as a standard component in UAV-based gas sensing missions and potentially be incorporated into emerging industry standards.

Counter-intuitively, tether length had no statistically significant effect on landing accuracy under low-wind conditions (1–2 m/s). This finding can be explained by the dominance of the UAV’s RTK-GPS hover accuracy (±0.1 m) over pendulum effects. The static pendulum angle due to wind drag is given by θ = arctan(F_wind_/mg), where F_wind_ = ½ρC_d_Av^2^_wind_, ρ is the air density, C_d is the drag coefficient, A is the projected area of the sensor, and v_wind is the wind speed. For the present load, the calculated lateral displacement is <0.11 m for a 14 m tether, which is negligible compared to the sensor’s post-landing bounce. However, this conclusion carries three important limitations that should guide future work and operational use.

First, wind speed sensitivity: the displacement scales with v_wind_^2^. At higher wind speeds, the pendulum displacement could reach 0.5–1 m, and tether length would then become a non-negligible factor. Second, sensor mass dependency: lighter sensors are more susceptible to wind-induced sway, and the negligible effect observed here for a 1 kg sensor may not hold for smaller payloads. Third, dynamic flight conditions: all tests were performed in static hover. In a real mission, the UAV may need to approach the target with forward velocity, causing the tether to swing like a pendulum. A settling delay of several seconds may be required to damp these oscillations before lowering the sensor. Future studies should therefore extend the present methodology to higher wind speeds, lighter sensors, and dynamic manoeuvres.

#### Limitations of the CFD Procedure

The CFD framework used in this study is adequate for estimating overall air velocity magnitudes and for guiding the design of the anti-downwash plate, but it has several limitations that should be acknowledged.

First, we used steady RANS (Reynolds-averaged Navier–Stokes) simulations, not URANS. Steady RANS assumes time-averaged flow and cannot capture transient vortex shedding, blade-passing frequency effects, or unsteady recirculation. This is a limitation for prop-wash flows, which are inherently unsteady and contain large-scale turbulent structures.

Second, the realizable k-ε model with enhanced wall treatment is widely used for engineering flows, but it is not optimal for swirling and separated flows. The standard k-ε model tends to over-predict turbulent kinetic energy in stagnation regions and under-predict the swirl intensity. For prop-wash under a multi-rotor UAV, the flow contains strong swirl, blade-tip vortices, and ground interaction. More advanced models such as RSM (Reynolds Stress Model), LES (Large Eddy Simulation), or DES (Detached Eddy Simulation) would provide better predictions of the swirling flow field, albeit at higher computational cost.

Third, despite these limitations, our CFD results were validated against field experiments using an ammonia source. The predicted 70% reduction in downwash velocity with the anti-downwash plate was confirmed experimentally, and the minimum safe tether length of 6 m was established based on both CFD and experimental data. This suggests that, for the purpose of estimating overall air velocity magnitudes and guiding plate design, the steady RANS k-ε approach was adequate.

Future work should use URANS or LES to capture unsteady prop-wash effects, especially for dynamic flight conditions, forward flight, and multi-rotor interference. Particle image velocimetry (PIV) experiments are also recommended for detailed validation.

### 4.3. Comparative Assessment of Deployment Strategies and Tactical Recommendations for Hazardous Chemical Scenarios

The three deployment strategies were compared across six normalised metrics (survival, accuracy, downwash resistance, mission time, terrain adaptability, and reusability), as visualised in the parallel coordinates plot and the Nightingale rose diagram. Tethered hovering achieved the highest composite scores: 100% survival, excellent downwash resistance (reading stability), strong terrain adaptability (can operate over rubble, vegetation, and slopes), and a moderate mission time of ≈3 min per point. Its main drawback is only moderate accuracy (dispersion diameter ≈ 1.7 m). Low-altitude placement offered perfect survival and superior accuracy (≤0.2 m) but required 8 min per point and flat, obstacle-free terrain, making it suitable only for fixed-point long-term monitoring on level surfaces. Direct drop, with 0% survival and poor reusability, is not viable without additional cushioning.

Based on these quantitative trade-offs, a mission-adaptive decision matrix was constructed for four representative hazardous chemical scenarios. Below, each scenario is described in detail, together with its core operational requirements and the recommended deployment strategy. For each scenario, we also indicate how UGVs or multi-sensor UAVs could complement the recommended strategy.

(1) Large-scale industrial accident (e.g., chemical plant fire or toxic leak)

In such incidents, the affected area often spans several square kilometres, and the terrain is typically complex and uneven due to collapsed structures, damaged pipelines, and storage tanks. Multiple hazardous substances may be present, and the time window for rescue and evacuation is extremely urgent. Environmental conditions can include smoke, heat radiation, and limited visibility. The core operational needs are a rapid wide-area survey to map contamination boundaries and plume dispersion, identification of the main toxic agents, and guidance for safe entry routes for ground responders—all while operating over debris without risking sensor loss. Tethered hovering is the sole viable option for this scenario. The UAV can maintain a safe altitude above 6 m while the sensor is lowered to the breathing zone to collect true exposure concentrations. The anti-downwash plate ensures data fidelity, and the sensor can be winched back and reused at multiple grid points. For a persistent leak source, such as a cracked storage tank, if a flat area is found nearby, low-altitude placement may be used as a secondary measure to leave a sensor for long-term unattended monitoring [26,27]. A UGV could also deploy the sensor on flat, accessible ground, while a multi-sensor UAV could map a wider area simultaneously.

(2) Terrorist attack in an urban environment (e.g., release of hazardous chemicals in subway vents, manholes, or building corners)

Urban settings are characterised by dense fabric that creates “urban canyon” effects, including GPS signal attenuation or multipath. Numerous potential concealment points exist, such as ventilation shafts, manhole covers, and underground parking entrances. High population density demands rapid confirmation of the presence and type of hazardous chemicals to guide evacuation and medical response, while minimising false positives to avoid panic. The operational priorities include rapid deployment to suspicious points, the ability to operate in GPS-denied or degraded environments, and precision placement into confined or partially obstructed spaces. Tethered hovering with augmentation is preferred. The UAV can hover above a manhole or ventilation grille while the sensor is lowered directly into the airflow from that point. For flat, accessible surfaces, low-altitude placement can be used to deposit a sensor for continuous monitoring, providing early warning of secondary release. A small UGV could access confined spaces or underground entrances that are difficult for UAVs. Direct drop is strictly prohibited in urban areas due to the risk of sensor ricochet, collateral damage, and uninterpretable landing poses [28].

(3) Hazardous chemical leakage from abandoned munitions (e.g., leaking containers in forested or hilly terrain)

These scenarios often involve low and intermittent emission rates, making the exact leak point difficult to localise. The terrain is typically natural—forests, hills, marshes—with uneven ground, vegetation, and limited accessibility for ground personnel. The hazardous chemicals may be denser than air, accumulating in low-lying areas. Therefore, the core needs are a wide-area search with high sensitivity to capture low-concentration plumes, the ability to sample very close to the ground (≤0.5 m) where heavy gases accumulate, long-duration trend monitoring once a hotspot is identified, and sensor reusability for repeated surveys over days or weeks [29]. A two-phase approach is recommended. In Phase 1 (search), tethered hovering is used in a grid pattern with the sensor lowered to 0.5 m above ground to maximise sensitivity for heavy gases. The UAV flies at a constant safe height of 6 m, and the winch rapidly lowers and raises the sensor at each waypoint. In Phase 2 (hotspot monitoring), if the terrain at the identified leak point is sufficiently flat (e.g., a clearing or a flat rock outcrop), low-altitude placement can deposit a sensor for long-term (hours to days) unattended operation, recording concentration trends without occupying the UAV. If the terrain is rough, tethered hovering can be used repeatedly at that point, leaving no sensor behind but requiring the UAV to remain on station [30,31,32,33,34]. A UGV could be used for ground-level sampling in accessible areas, and a multi-sensor UAV could increase search throughput.

(4) Hazardous material transport accident (e.g., tanker rollover on a highway slope or in a ditch)

Accident locations are random and often occur on sloped surfaces such as road embankments, highway medians, or drainage ditches, with obstacles including guardrails, vehicle wreckage, and spilled cargo. The terrain is highly uneven and may be contaminated with flammable or corrosive substances. Time pressure is extreme, as any delay may allow the plume to reach populated areas downstream. The operational needs are immediate aerial reconnaissance to assess the spill extent and plume direction, the ability to place a sensor close to the leaking container (e.g., near a tanker valve or a pooling area) without landing the UAV, avoidance of sensor damage or entanglement, and rapid mission turnaround. Tethered hovering is the only feasible method. The UAV can safely hover above obstacles while the sensor is lowered on the tether to the desired height (e.g., 0.5 m above the leak source). Low-altitude placement is impossible on a steep or irregular slope, and direct drop would almost certainly destroy the sensor. The winch system also features a remote-cut-off function: if the sensor becomes entangled or contaminated with sticky residue, the tether can be severed, sacrificing the sensor but preserving the much more expensive UAV platform. This risk-mitigation feature is particularly valuable in transport accidents where the chemical may be unknown or particularly aggressive [35]. A UGV could approach the site from a safe direction on accessible ground, but its mobility may be limited by slopes and debris.

In summary, tethered hovering with downwash mitigation is recommended as the primary deployment strategy for most complex hazardous chemical scenarios owing to its high survival rate, excellent data reliability, strong terrain adaptability, and reusability. Low-altitude placement serves as a complementary method for specific flat-surface long-term monitoring tasks where centimetre-level precision is required. Direct drop should be avoided altogether until lightweight cushioning technologies are developed and validated. UGVs and multi-sensor UAVs can further expand the operational envelope, especially for flat-surface placement, confined-space access, and wide-area coverage. The decision matrix presented in Figure 14 provides tactical commanders with an evidence-based tool for rapid, on-site selection of the optimal deployment method, directly addressing the time-sensitive nature of hazardous chemical emergencies.

## 5. Conclusions

This study systematically evaluated three deployment strategies for a UAV-based chemical sensor through controlled field experiments, CFD simulations, and multi-dimensional performance comparison. Direct free-fall drop from 5 m resulted in 0% survival due to impact-induced shell fracture and component detachment, rendering it infeasible without lightweight cushioning (e.g., EPP foam) and height reduction to ≤3 m. A carbon-fibre anti-downwash plate was shown to reduce local airflow velocity by ≈70%, establishing a minimum safe tether length of 6 m and ensuring measurement fidelity. Tethered hovering achieved 100% survival, excellent terrain adaptability, and a mission time of ≈3 min per point, making it the recommended primary strategy for most hazardous chemical scenarios. Low-altitude gentle placement offered perfect survival and sub-decimetre accuracy but required 8 min per point and flat, obstacle-free surfaces, limiting its use to fixed-point long-term monitoring.

Based on these experimental results, we derived a mission-adaptive decision matrix that maps the three strategies to four typical chemical threat scenarios—industrial accidents, urban terrorist attacks, abandoned munition leakage, and transport incidents—with clear actionable recommendations. This framework directly addresses the time-sensitive nature of emergency responses by enabling rapid, on-site selection of the optimal deployment method according to mission priorities and terrain conditions. While the present study focuses on a single-sensor UAV platform, the framework can be extended to multi-sensor UAVs, UGVs, and UAV–UGV collaborative systems. Future work should validate these extensions experimentally and explore multi-agent deployment planning. Beyond the specific findings, our work shifts the discourse from whether unmanned systems can carry chemical sensors to how to deploy them reliably, efficiently, and adaptively in the field—a transition that has been frequently discussed but rarely supported by systematic experimental validation. We anticipate these results will accelerate the adoption of unmanned chemical reconnaissance in routine emergency operations and inform future design standards for impact-resistant sensor packaging and downwash-mitigating accessories.

## Figures and Tables

**Figure 1:**
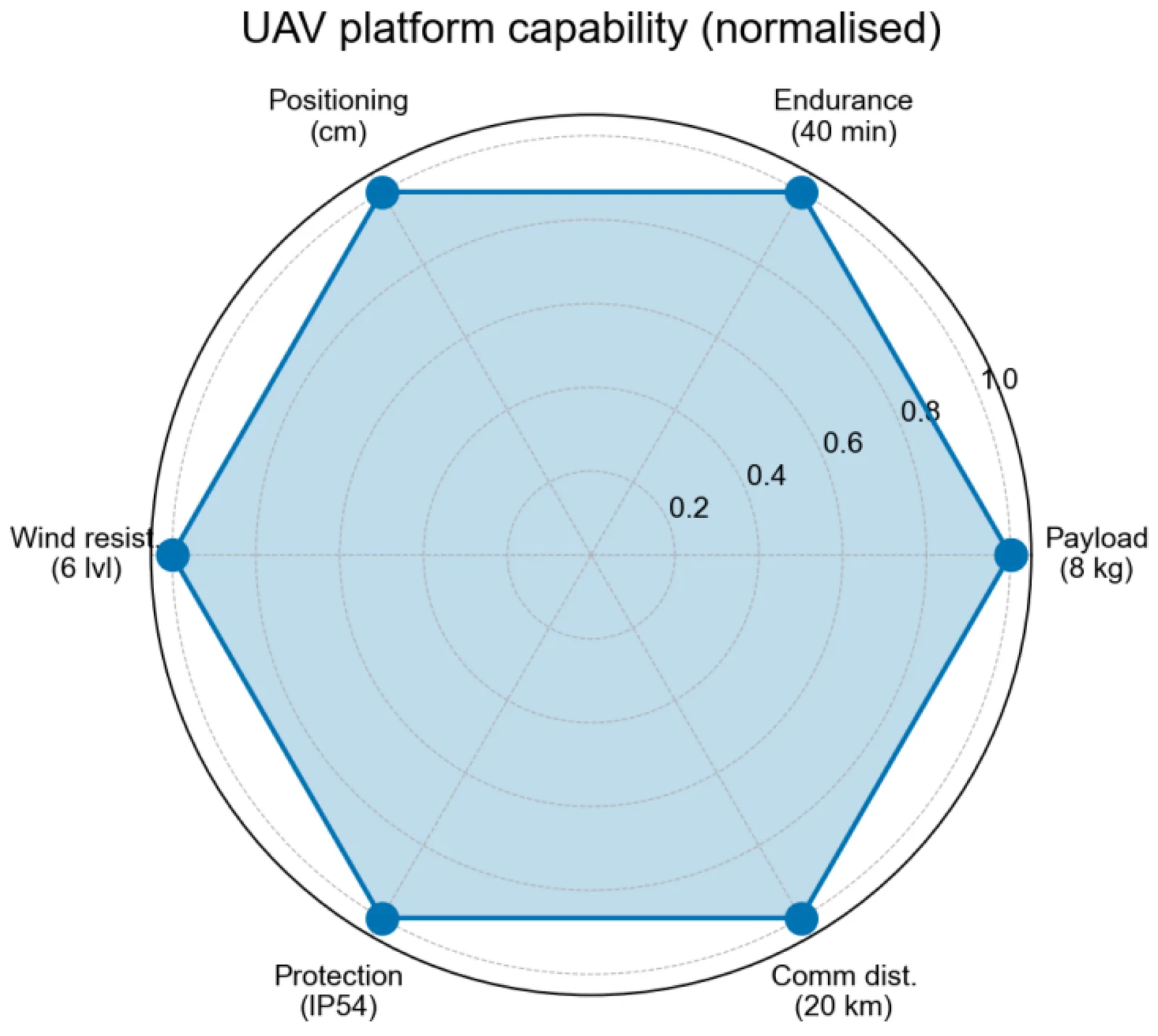
UAV platform capability radar chart.

**Figure 2:**
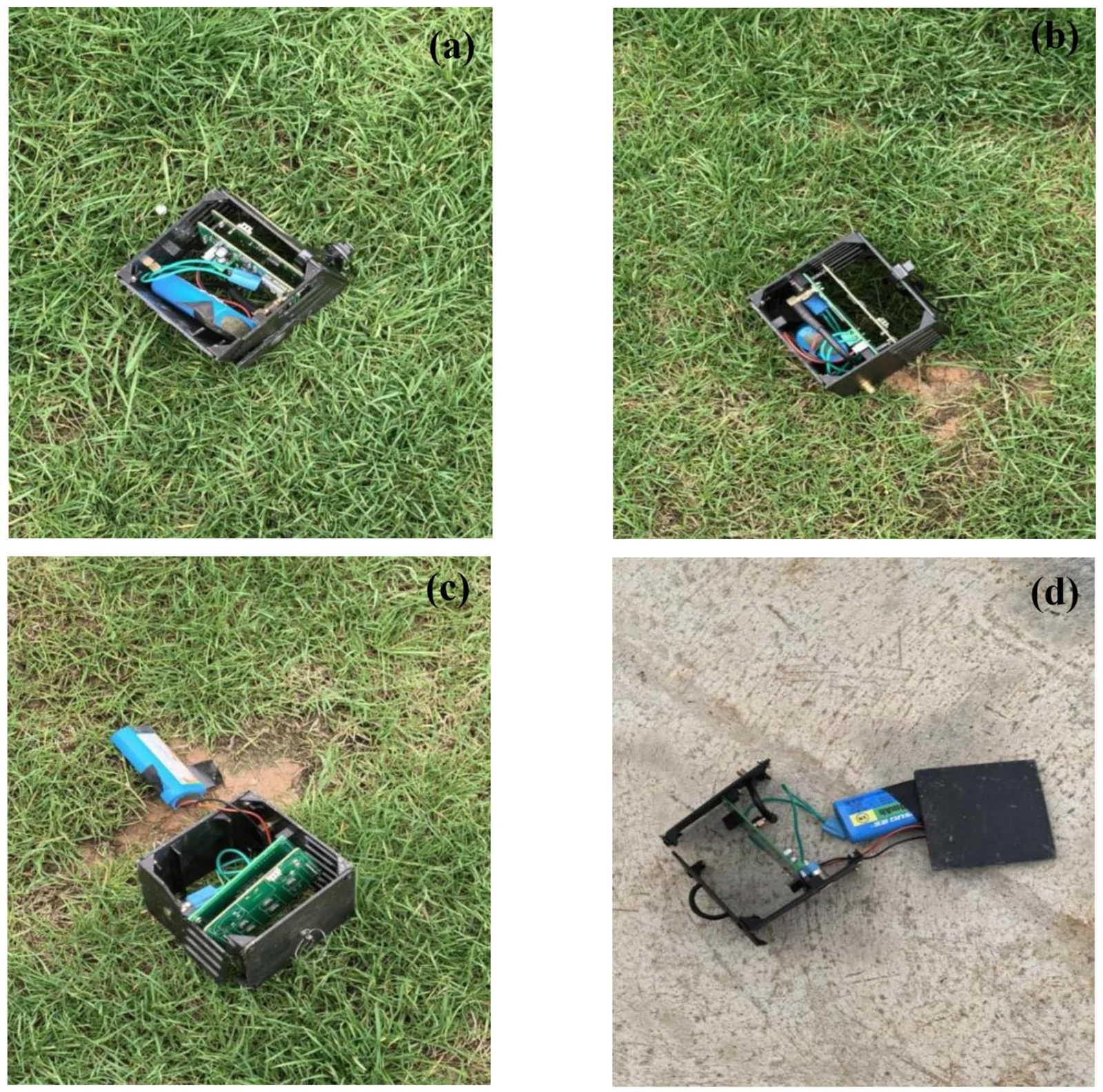
Sensor damage after 5 m free-fall drop: (**a**) concrete surface—complete shell rupture; (**b**) concrete surface—power switch detachment; (**c**) grass surface—severe damage; (**d**) grass surface—minor crack with functional reassembly. The UAV hovered at 5 m before release.

**Figure 3:**
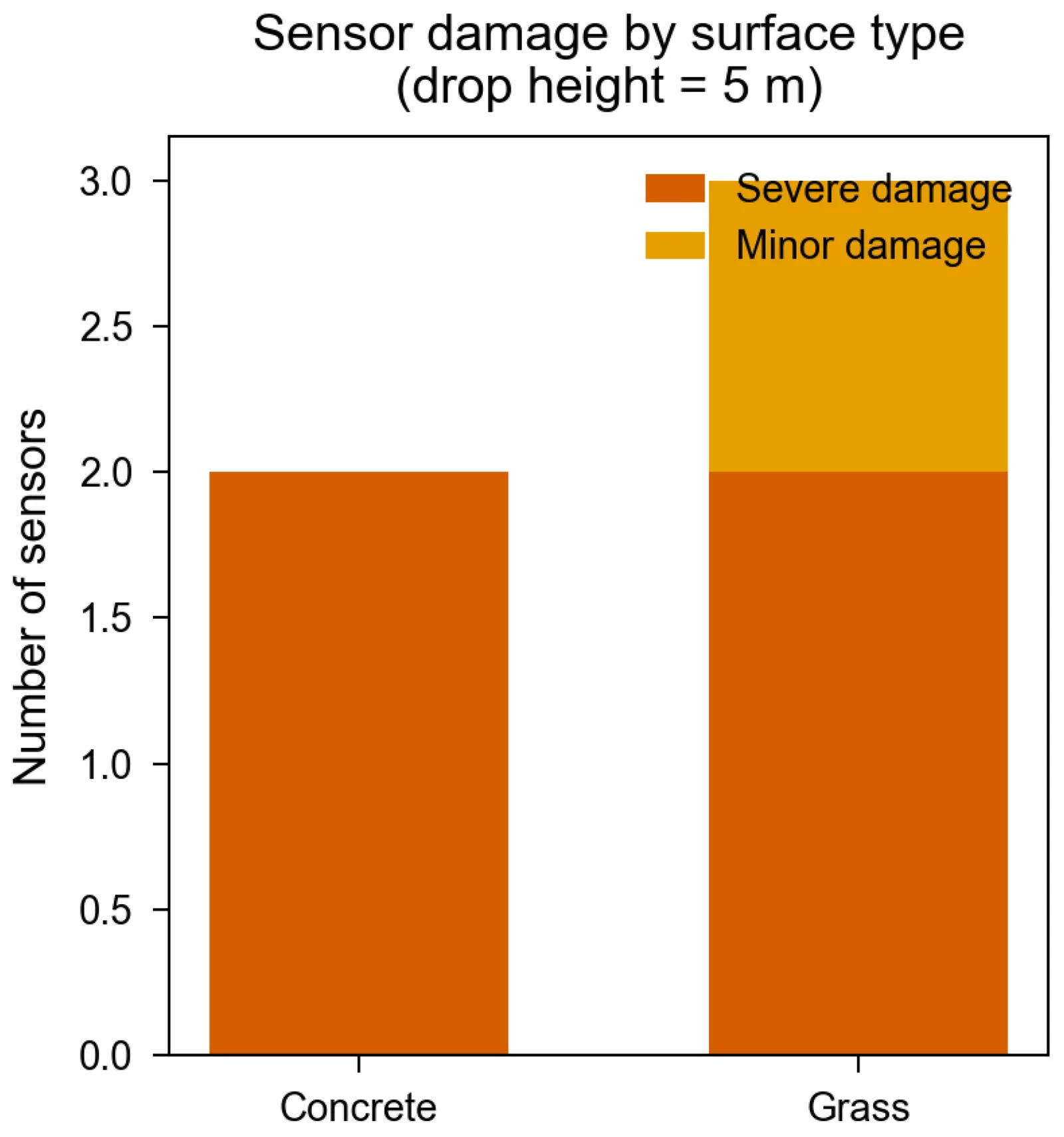
Stacked bar chart of sensor damage severity by surface type.

**Figure 4:**
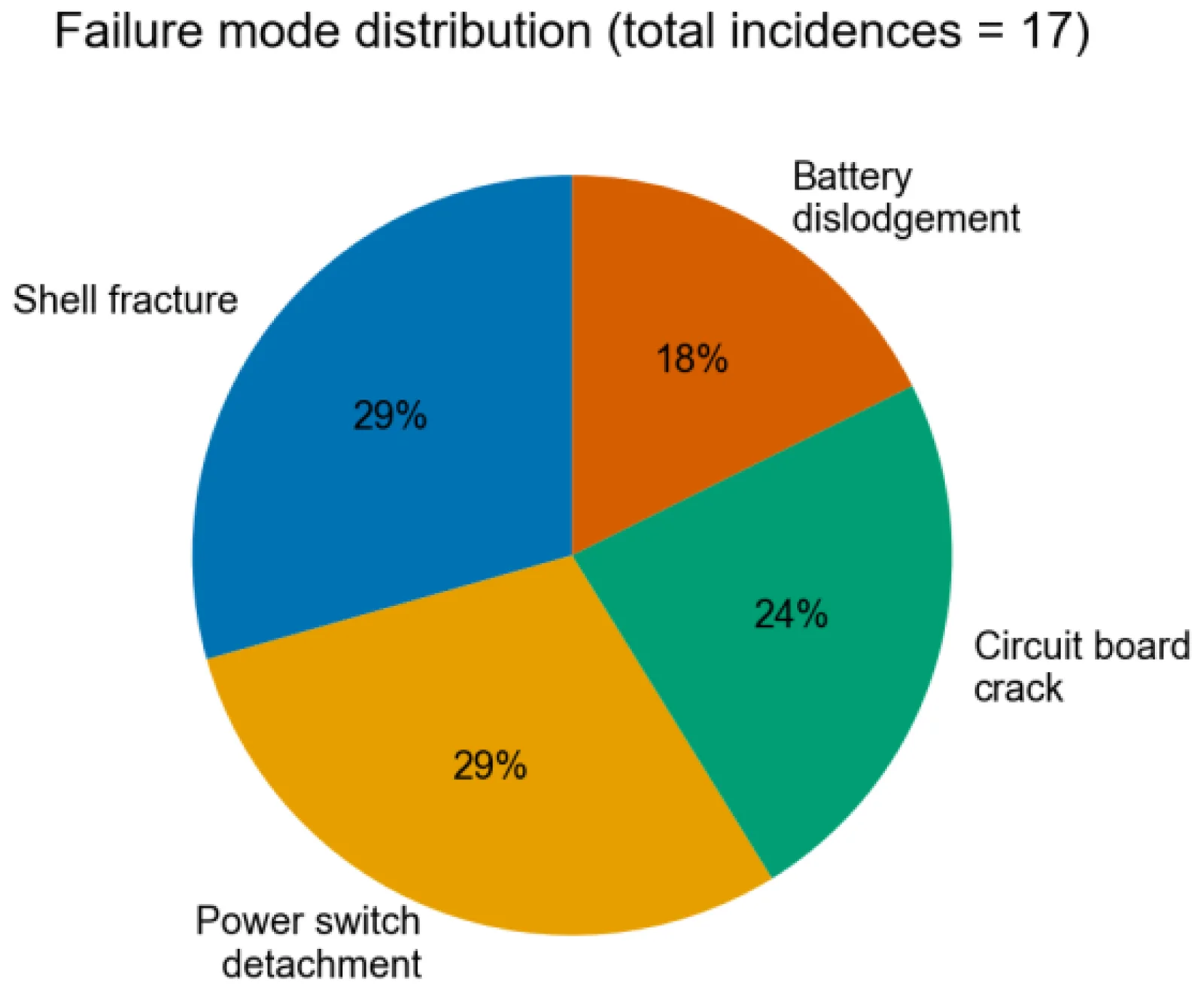
Pie chart of failure mode distribution.

**Figure 5:**
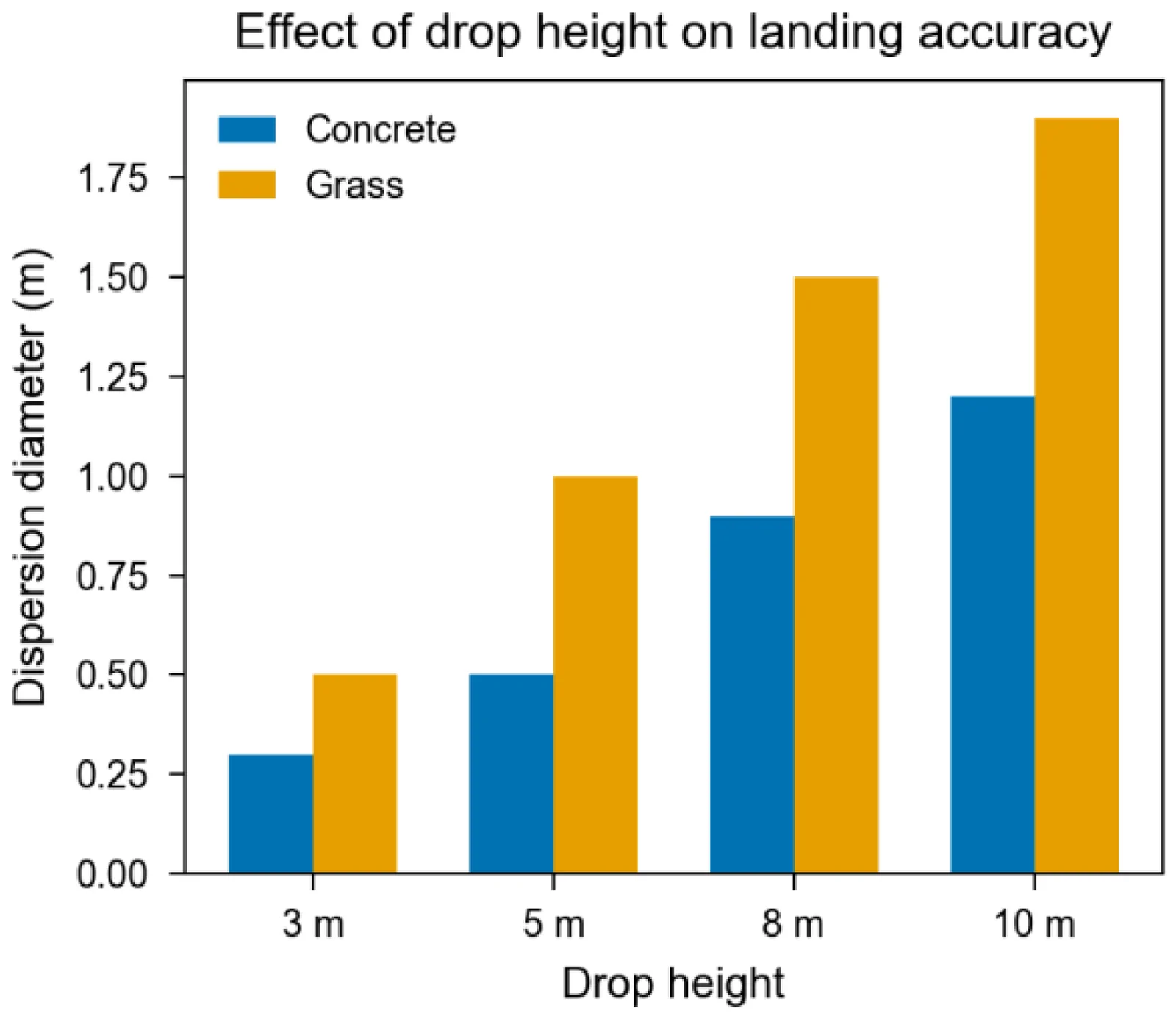
Effect of drop height on landing accuracy: dispersion diameter vs. drop height.

**Figure 6:**
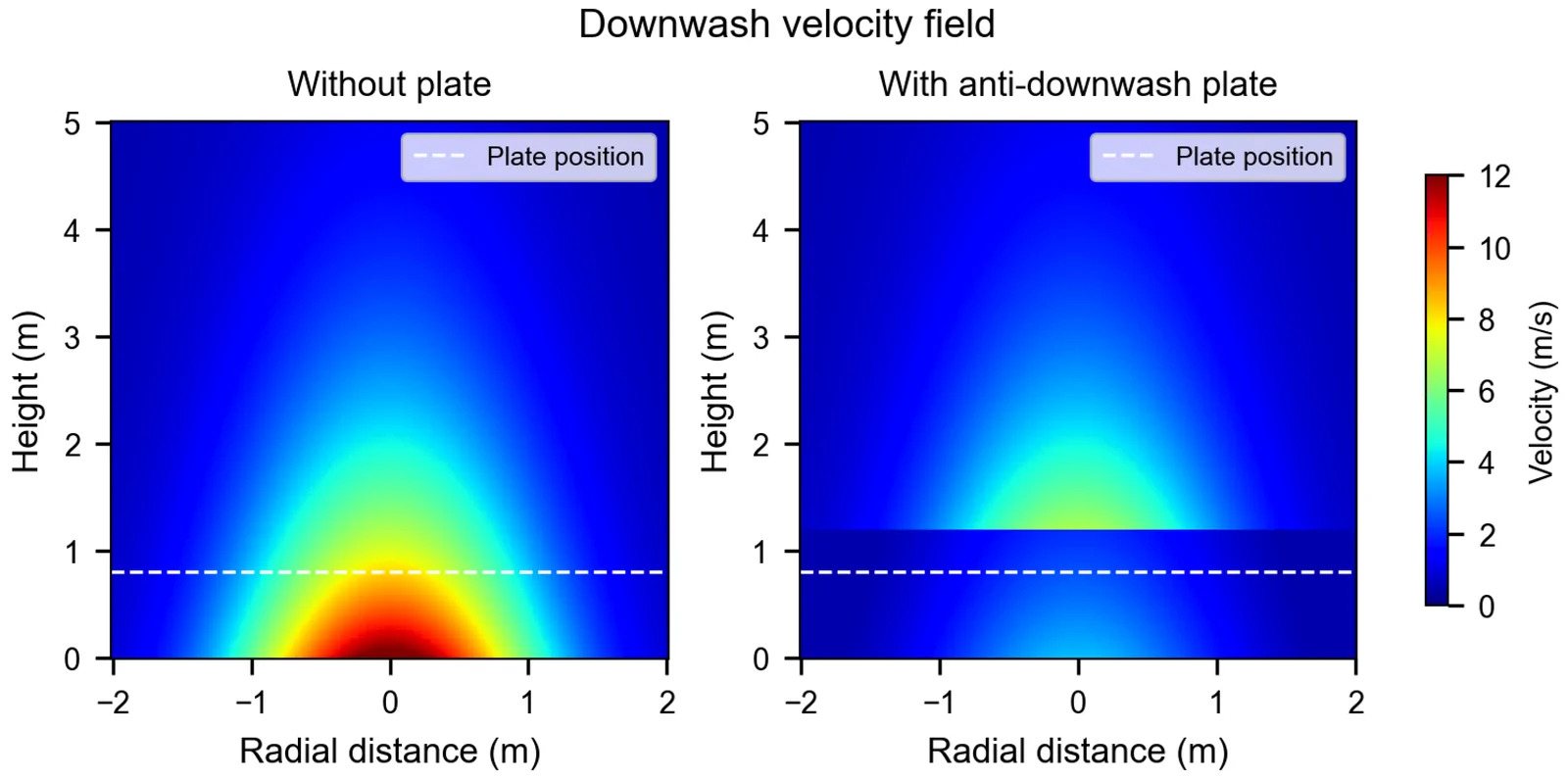
CFD simulation of downwash velocity field (contour plot).

**Figure 7:**
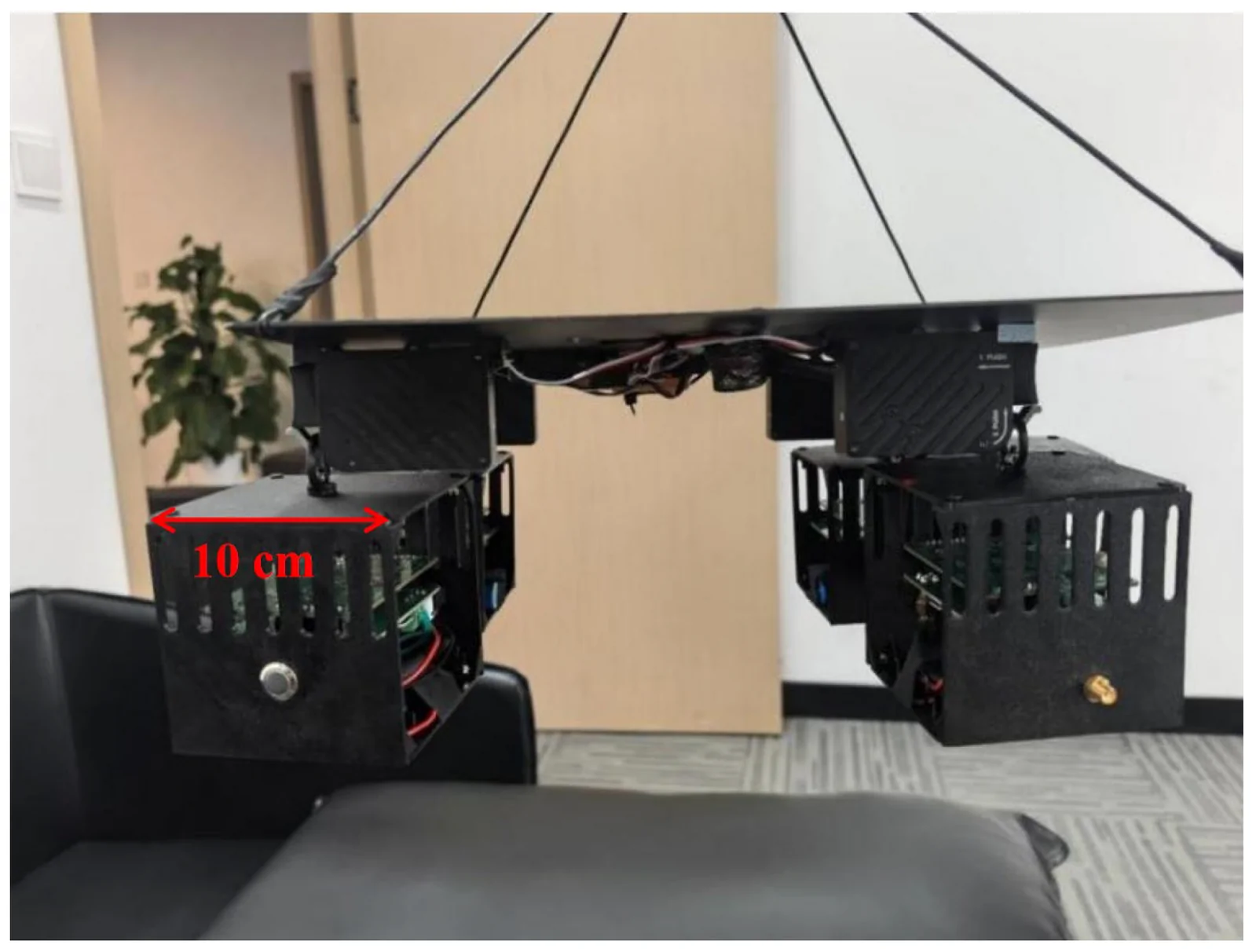
Photograph of the installed assembly of the anti-downwash plate, with a scale bar (10 cm). The plate diameter is 380 mm, the suspension rods are 150 mm in length, and the sensor is suspended 120 mm below the plate.

**Figure 8:**
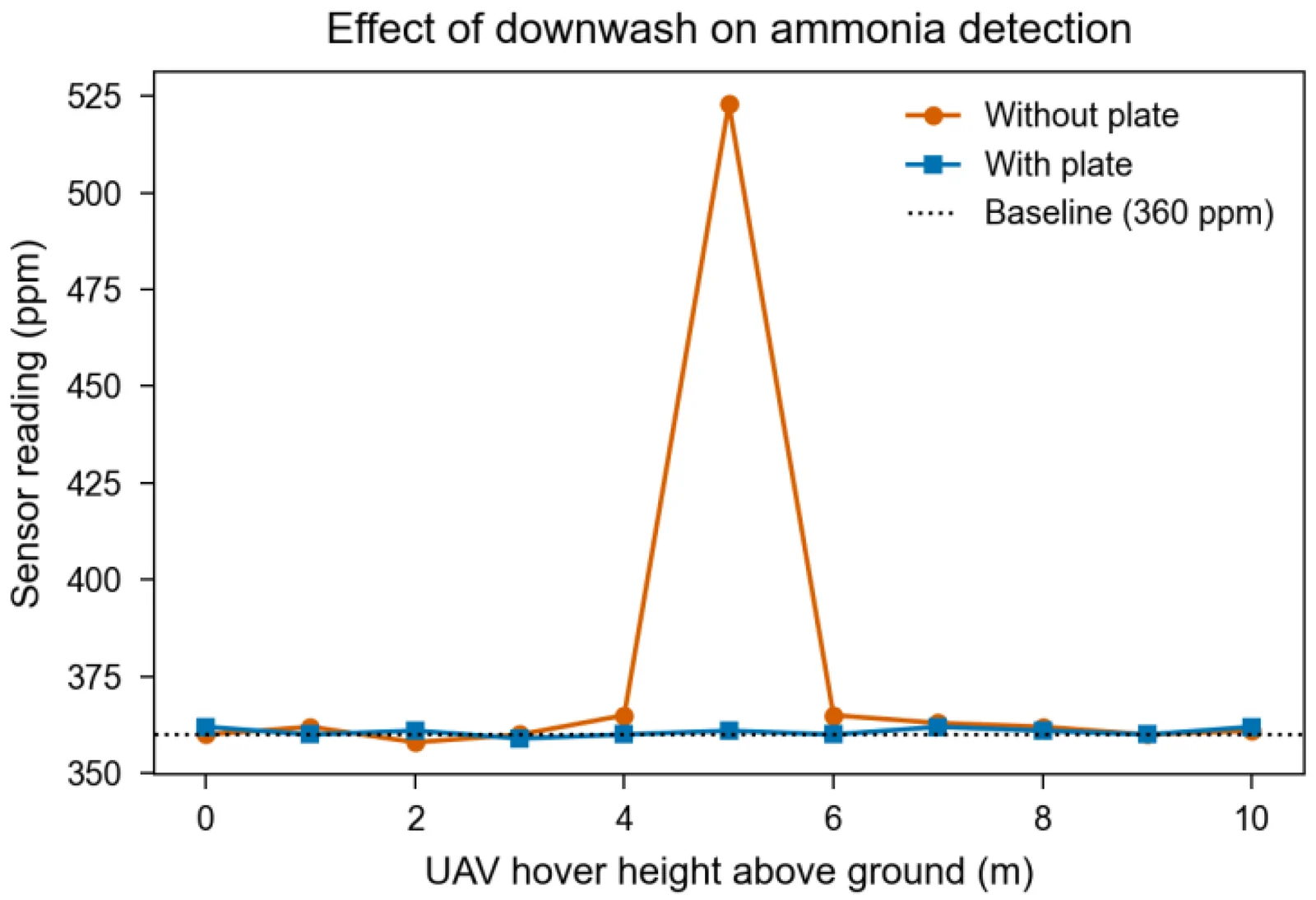
Downwash interference on sensor readings: with vs. without anti-downwash plate.

**Figure 9:**
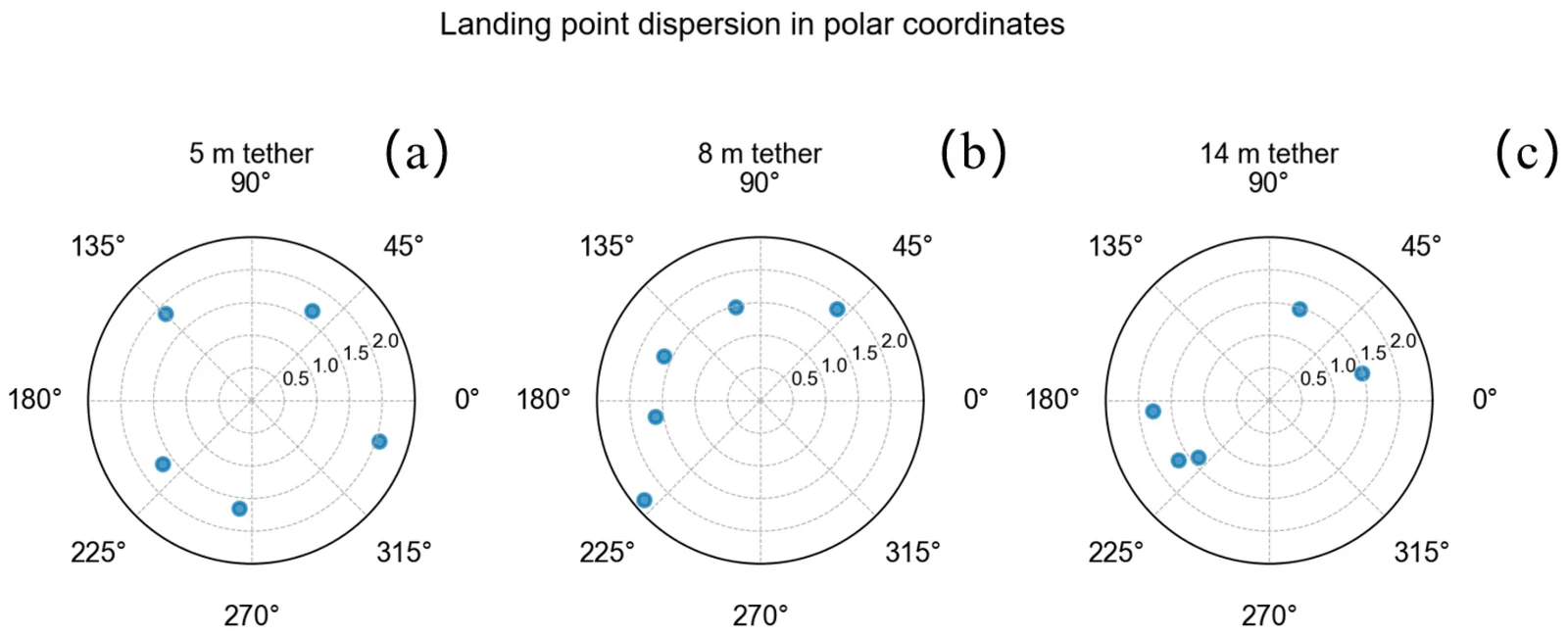
Landing point dispersion in polar coordinates for different tether lengths: (**a**) 5 m tether; (**b**) 8 m tether; (**c**) 14 m tether.

**Figure 10:**
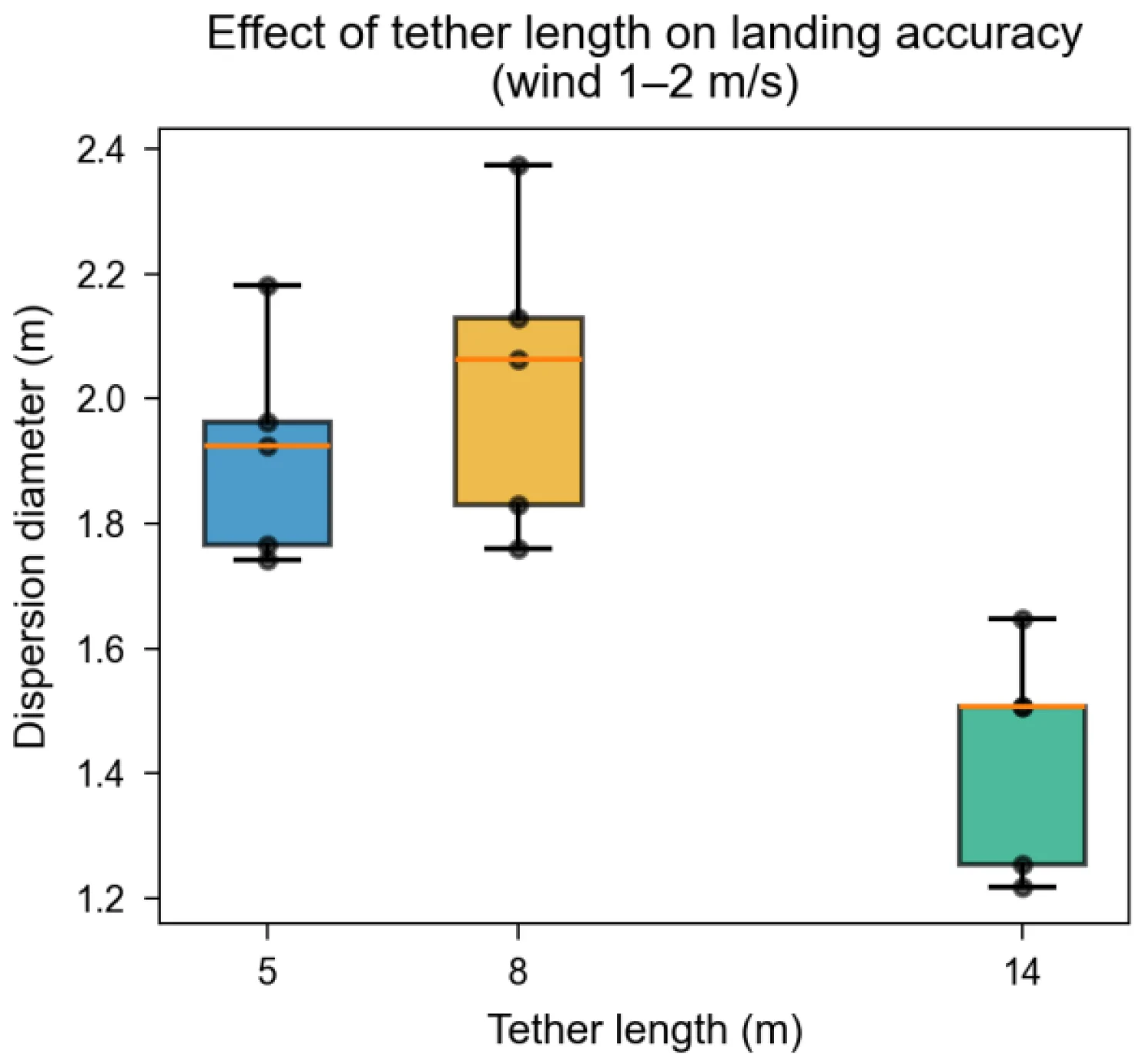
Box-and-whisker plot of landing dispersion vs. tether length.

**Figure 11:**
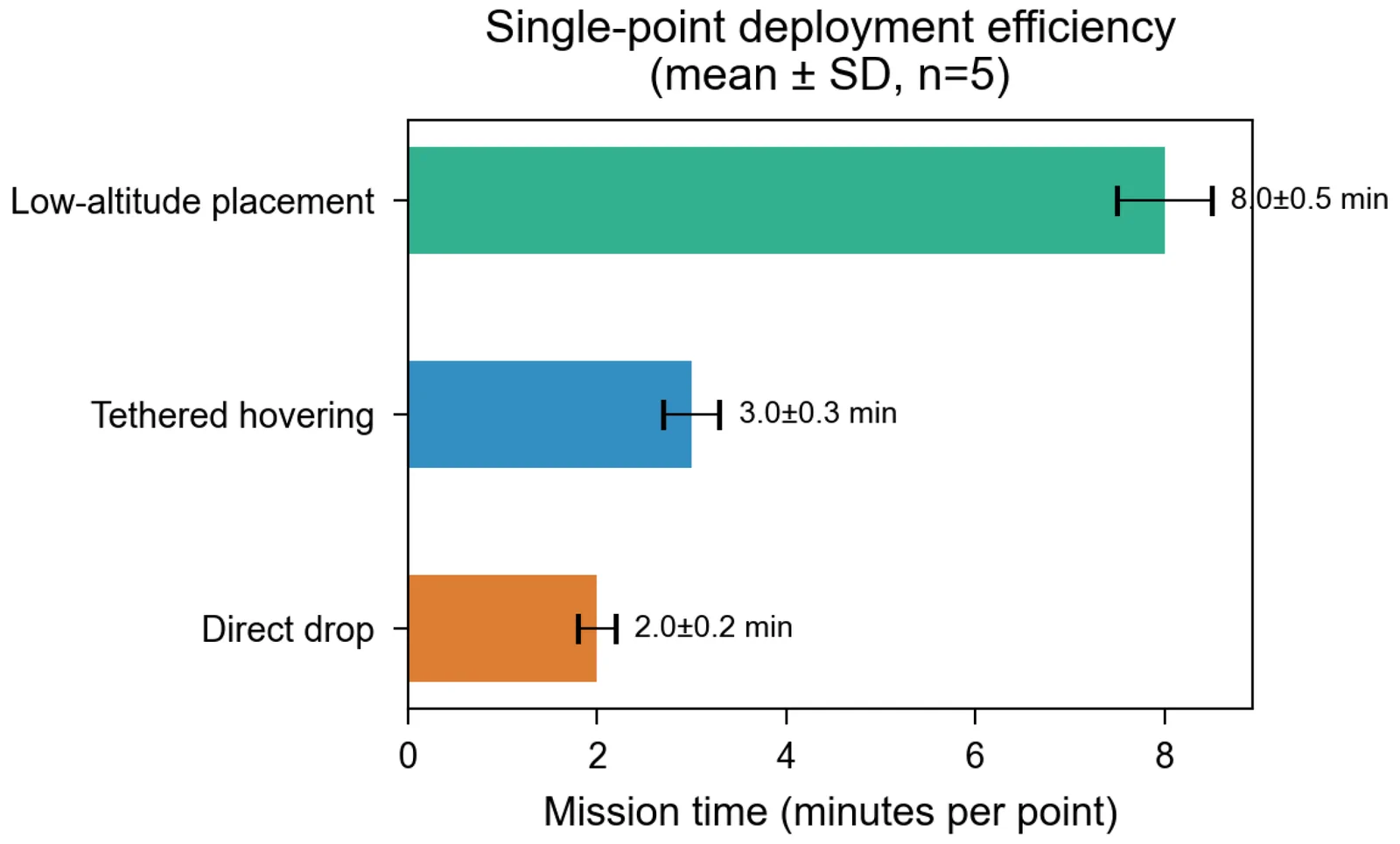
Mission time comparison among three deployment strategies (mean ± SD, n = 5).

**Figure 12:**
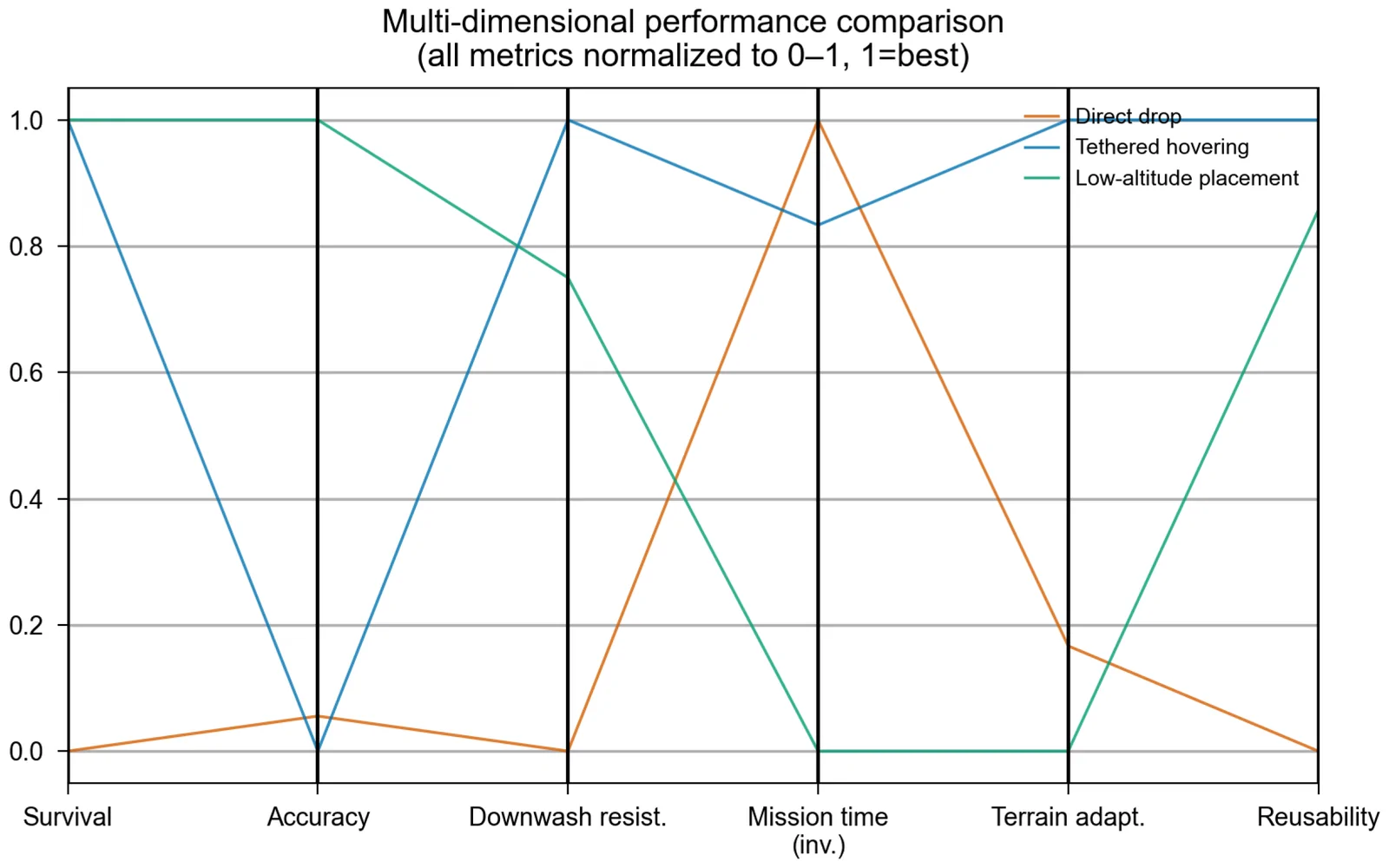
Parallel coordinates plot for multi-dimensional performance comparison.

**Figure 13:**
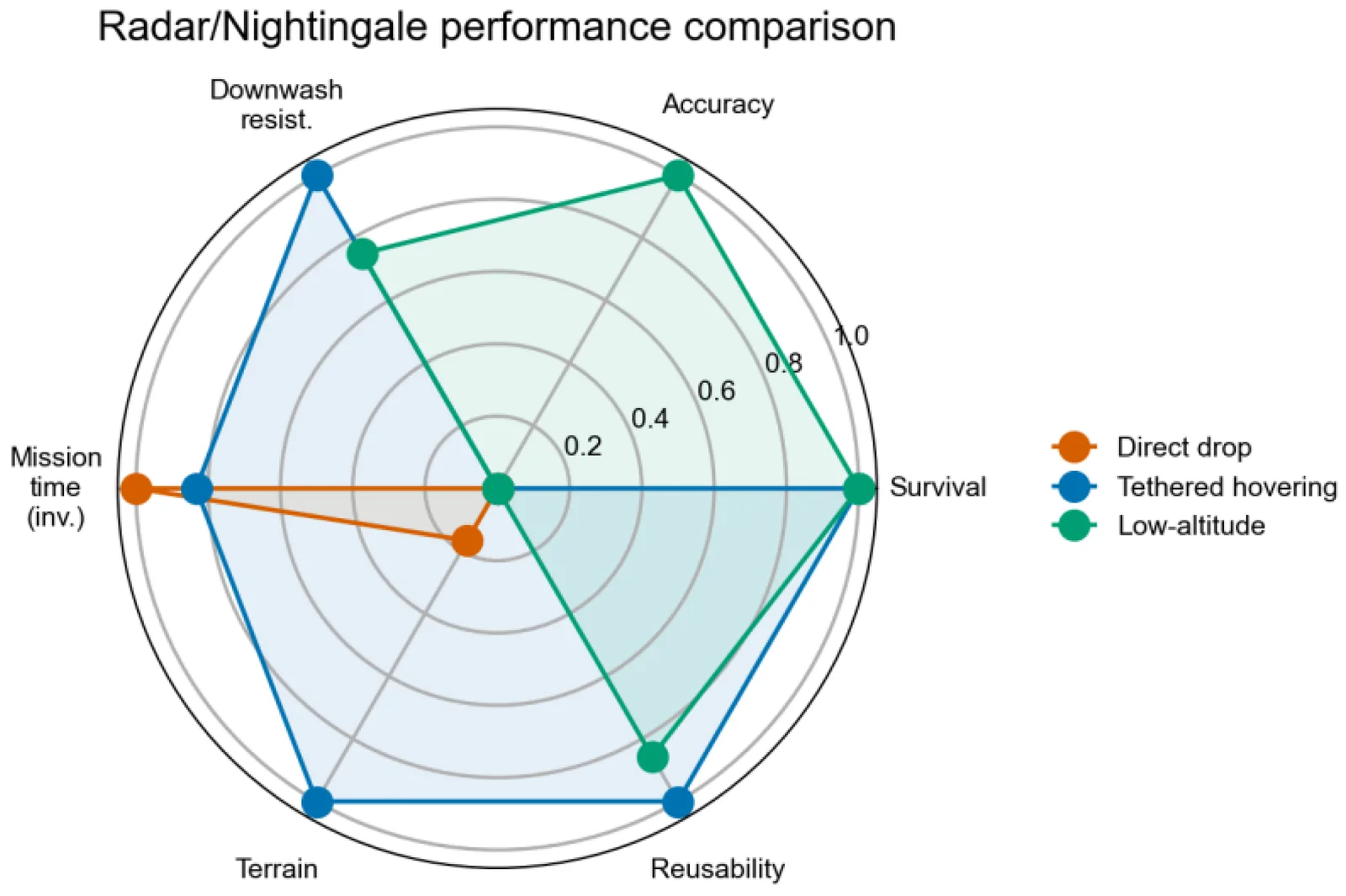
Nightingale rose diagram (polar radar chart) of three deployment strategies.

**Figure 14:**
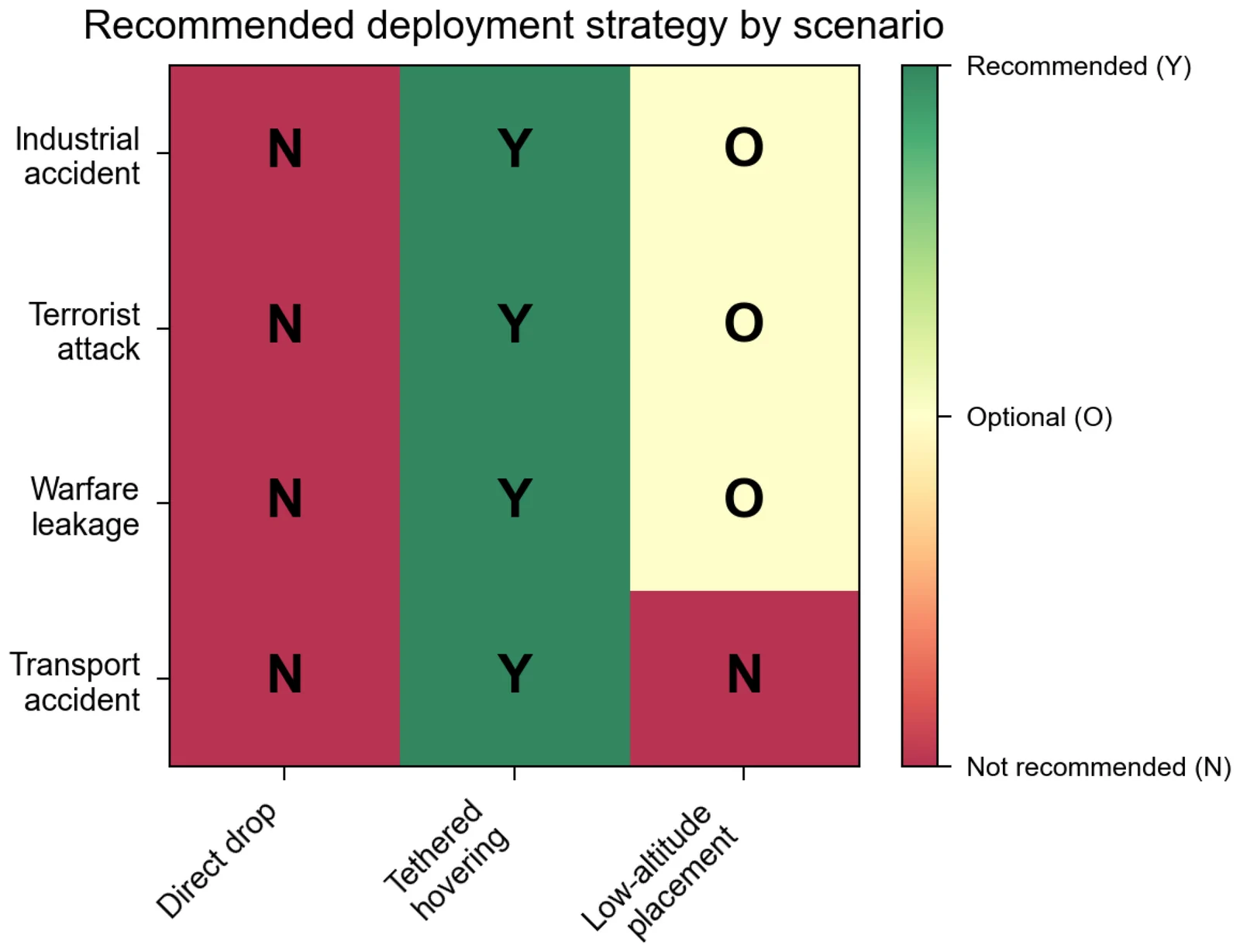
Decision matrix heatmap: recommended deployment strategies for four chemical threat scenarios.

**Table 1:** Estimated survival probability and mass penalty for advanced enclosure designs at 5 m drop.

Design	Estimated Survival	Mass Penalty	Notes
Baseline ABS (2.5 mm)	0%	–	Current design
ABS + 10 mm EPP foam	>80%	~50 g	Lightweight cushioning
CFRP shell + internal suspension	>95%	~150 g	Higher cost, reusable
Active airbag system	>98%	~120 g	Complex, for expensive sensors
Controlled-opening package box	>90%	~100 g	Opens after landing

## Data Availability

The experimental data supporting the findings of this study (sensor survival rates, landing dispersion coordinates, downwash readings, and CFD simulation results) are available from the corresponding author upon reasonable request. No publicly archived datasets were used in this study.

## Supplementary Materials

The following supporting information can be downloaded at: https://www.mdpi.com/article/10.3390/s26216985/s1, Figure S1: Actual images of the drone used in this study; Figure S2: The total weight of the modified sensor and carbon plate.
