# Supplementary material for: Field Deployment Strategies for UAV-Based Chemical Sensors: Systematic Evaluation of Direct Drop, Tethered Hovering, and Low-Altitude Placement in Hazardous Chemical Scenarios
Source: Sensors (Basel). 2026 Nov 5;26(21):6985. doi: 10.3390/s26216985 (PMC13766357; doi:10.3390/s26216985)
Supplement: Supplementary file 1 [file sensors-26-06985-s001.zip › sensors-4554082-supplementary.pdf]

Supplementary Materials

Photocatalytic Flow-Through CDI for Water-Salt Synergy via Detoxification and Clean Brine Generation Enabling Salt Recovery from High-Salinity Wastewater

Institute of NBC Defence PLA ARMY, Beijing, China
* Correspondence: 18511090259@163.com; Tel.: +86-18511090259

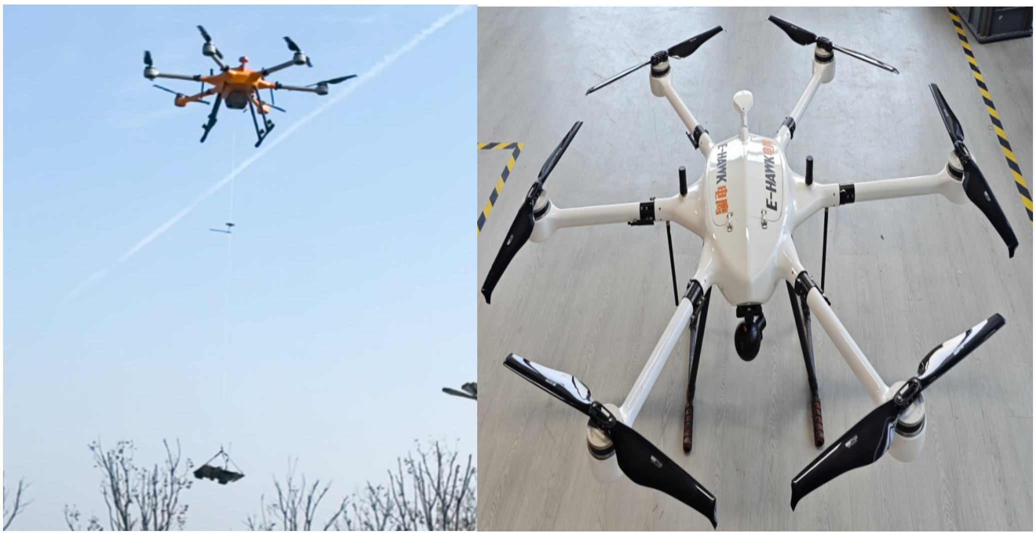

Figure S1. Actual images of the drone used in this study.

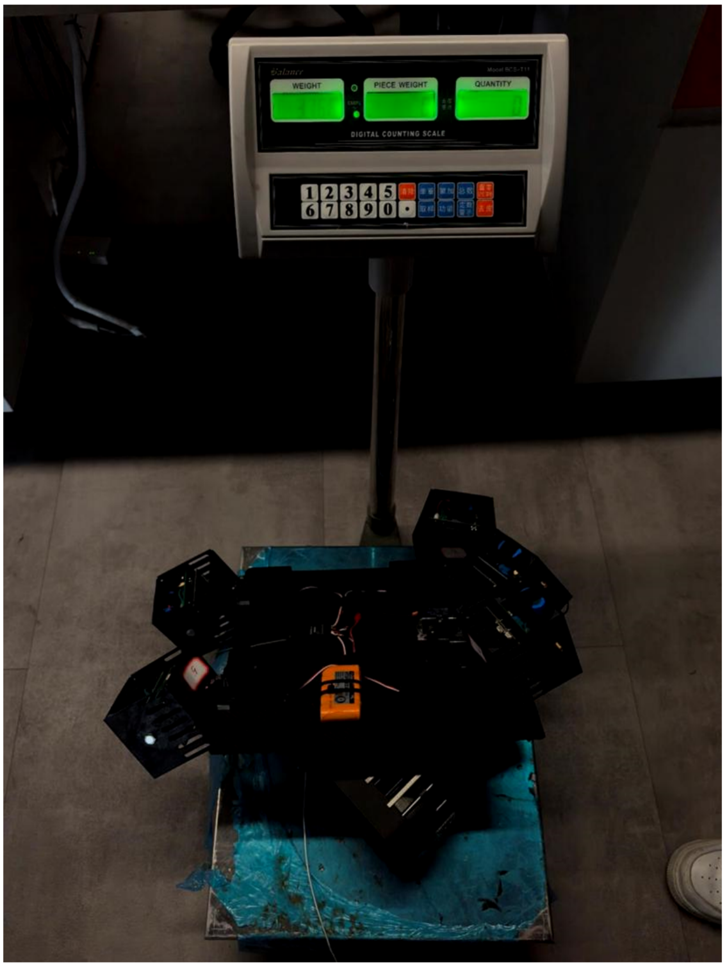

Figure S2. The total weight of the modified sensor and carbon plate.
